# Perioperative risk scores: prediction, pitfalls, and progress

**DOI:** 10.1097/ACO.0000000000001445

**Published:** 2024-11-20

**Authors:** Jonathan P. Bedford, Oliver C. Redfern, Benjamin O’Brien, Peter J. Watkinson

**Affiliations:** a Kadoorie Centre for Critical Care Research and Education, Nuffield Department of Clinical Neurosciences, University of Oxford, Oxford; b Milton Keynes University Hospital NHS Foundation Trust, Milton Keynes, UK; c Department of Cardiac Anesthesiology and Intensive Care Medicine, Deutsches Herzzentrum der Charité, Charité Universitätsmedizin Berlin, Berlin, Germany; d Department of Perioperative Medicine, St. Bartholomew's Hospital and Barts Heart Centre, Barts Health NHS Trust, London; e Oxford University Hospitals NHS Foundation Trust, Oxford, UK

**Keywords:** perioperative, prognostic modelling, risk scores, Surgery

## Abstract

**Purpose of review:**

Perioperative risk scores aim to risk-stratify patients to guide their evaluation and management. Several scores are established in clinical practice, but often do not generalize well to new data and require ongoing updates to improve their reliability. Recent advances in machine learning have the potential to handle multidimensional data and associated interactions, however their clinical utility has yet to be consistently demonstrated. In this review, we introduce key model performance metrics, highlight pitfalls in model development, and examine current perioperative risk scores, their limitations, and future directions in risk modelling.

**Recent findings:**

Newer perioperative risk scores developed in larger cohorts appear to outperform older tools. Recent updates have further improved their performance. Machine learning techniques show promise in leveraging multidimensional data, but integrating these complex tools into clinical practice requires further validation, and a focus on implementation principles to ensure these tools are trusted and usable.

**Summary:**

All perioperative risk scores have some limitations, highlighting the need for robust model development and validation. Advancements in machine learning present promising opportunities to enhance this field, particularly through the integration of diverse data sources that may improve predictive performance. Future work should focus on improving model interpretability and incorporating continuous learning mechanisms to increase their clinical utility.

## INTRODUCTION

Clinical prediction models aim to estimate an individual's probability of current (a diagnostic model) or future (a prognostic model) events. Multiple models have been developed to estimate the risk to patients of perioperative complications and death [1]. These perioperative risk scores have been used to inform preoperative assessment, surgical planning, and patient counselling [2]. Their use can inform communication with patients and promote more individualized perioperative care [3].

Many tools are available but need to be understood in the context of their intended use and limitations. Older tools have required updating to ensure reliability of risk estimates, and modern machine learning techniques are being developed but have yet to be robustly validated.

In this review, we introduce common model performance metrics and pitfalls in model development. We review common perioperative risk scores [1], discuss their limitations, and explore recent advances and future directions in perioperative risk modelling. 

**Box 1:**
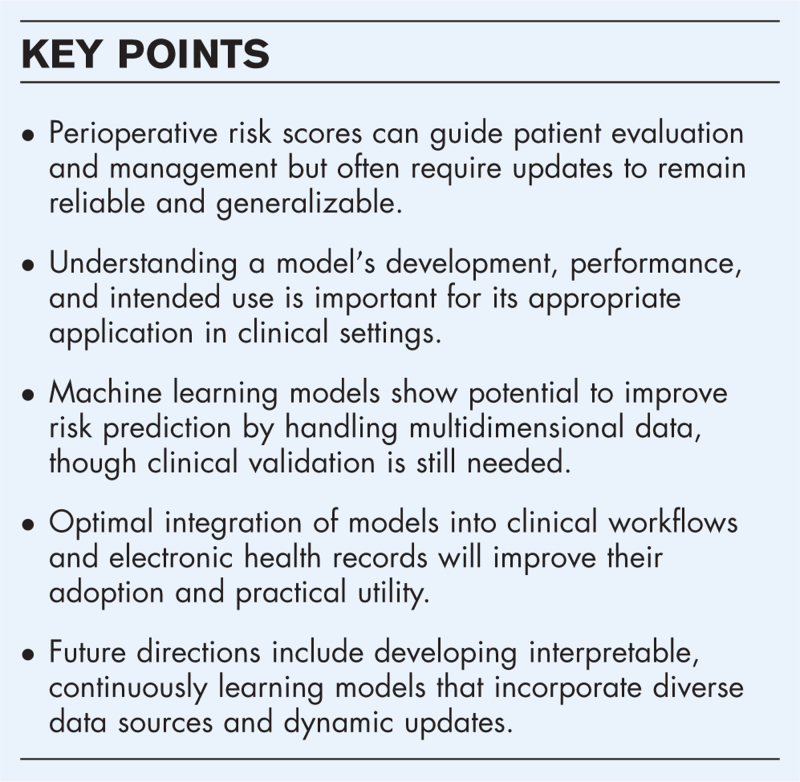
no caption available

## ASSESSING MODEL PERFORMANCE

Prediction modelling is concerned with predicting an outcome based on certain variables or predictors. Perioperative risk scores are a practical example of this process, and their utility depends on their ability to reliably predict outcomes. Prediction models require robust performance evaluation prior to use in clinical practice. Key metrics for this purpose are explored below, focusing on the concepts of discrimination and calibration.

### Discrimination

Discrimination measures a model's ability to assign a higher predicted risk to patients who experience an event compared to those who do not. The concordance statistic (*c*-statistic) is a commonly used metric of discrimination, representing probability that a randomly selected patient who developed the outcome of interest has a higher predicted risk than a patient who did not develop the outcome. The c-statistic ranges from 0 to 1, with 0.5 representing a model with no more predictive power than a random guess. In a model predicting binary events, the *c*-statistic is equivalent to the area under the receiver-operating characteristic (ROC) curve (AUROC). The ROC curve is a plot of the sensitivity (true positive rate) against the false positive rate across the full range of risk thresholds.

The *c*-statistic is a summary of a model's discriminative ability; however it does not describe sensitivity or specificity at different risk thresholds. This information can be obtained from a labelled ROC curve. Furthermore, the c-statistic provides no insight into whether the overall magnitude of risk is predicted accurately. This aspect is measured by a model's calibration metrics.

### Calibration

Clinical decisions are based on predicted risks, so these risks should be reliable. A model may have good discrimination, but provide inaccurate absolute risk estimates, either for all patients or those in specific subgroups.

**FIGURE 1:**
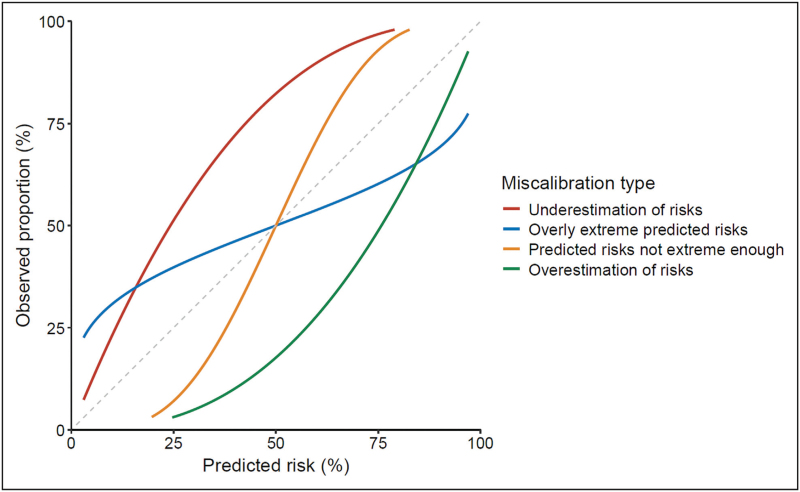
Examples of different types of miscalibration. Poorly calibrated models may systemically underestimate (red line) or overestimate (green line) risks. Models may also produce estimates of risk that are too extreme (blue line) or too modest (yellow line).

Overall calibration (“calibration-in-the-large”) compares the average predicted risk with the overall event rate [4]. Where observed proportions are higher than the predicted probabilities, calibration-in-the large >0, and vice versa (Figure 1; red and green lines, respectively).

Whether the “spread” of predicted probabilities is appropriate can be assessed with the calibration slope [5]. A model with extreme predictions (too low and too high) will have a calibration slope *<*1. It may provide reliable risks for patients at medium risk, but may, for example, suggest a near-100% risk of an outcome when the observed risk in that group is 75%, or predict a near-0% risk in patients where the event is observed in 25% (Fig. 1; blue line). Conversely models may produce modest predictions (not high enough nor low enough), demonstrated by a calibration slope > 1 (Fig. 1; yellow line). Well calibrated models may therefore be more clinically useful than poorly calibrated models with higher discrimination.

**FIGURE 2:**
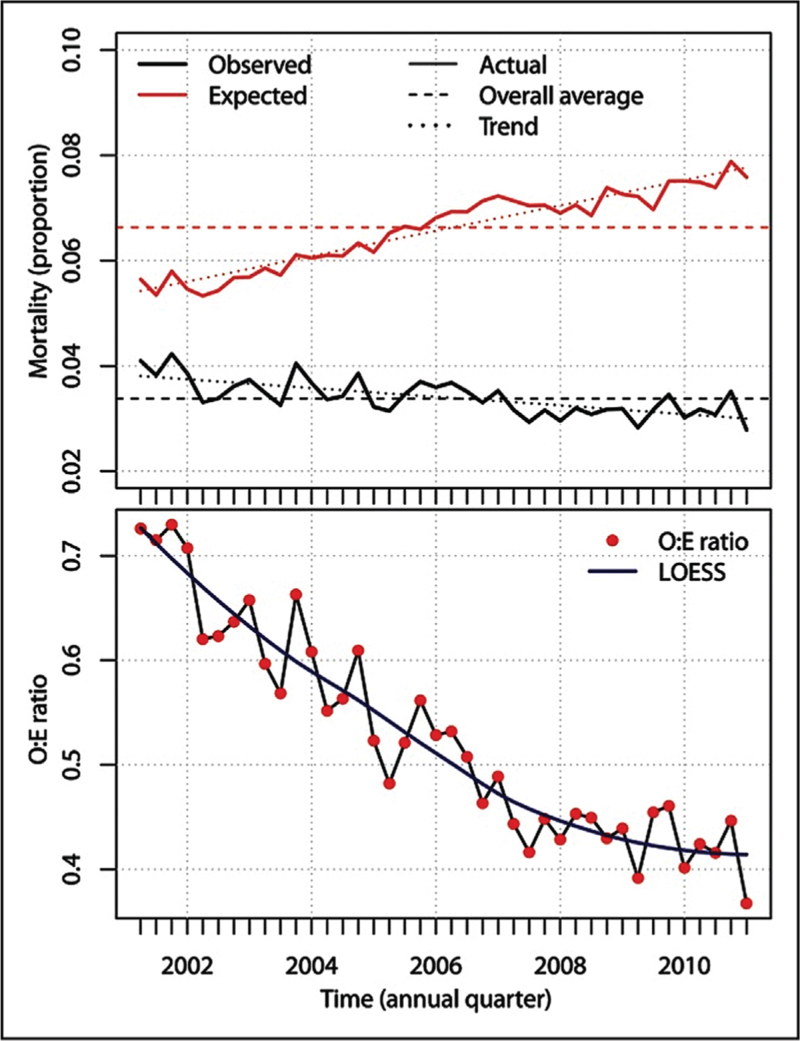
Calibration drift. O:E ratio, ratio or observed to expected mortality; LOESS, locally estimated scatterplot smoothing. Figure reproduced with permission from Hickey *et. al.*[6].

As populations and interventions change, models may require recalibration to maintain the accuracy of predictions. Recalibration can take many forms, from re-estimating a model intercept through to re-fitting a model entirely (re-estimating each coefficient, with or without adjusting the included predictors). As an example, the EuroSCORE model (a perioperative risk score for patients undergoing cardiac surgery) was found to demonstrate “calibration drift”, where calibration worsens over time as populations and practices change from those on which the model was developed, resulting in predicted mortality being more than double that of observed mortality (Figure 2) [6]. The original EuroSCORE variables were modified to produce the EuroSCORE II model, showing much improved calibration [7].

## LIMITATIONS AND PITFALLS OF CLINICAL PREDICTION SCORES

Some of the key challenges in the development of clinical prediction models are outlined below.

### Overfitting

As models become more complex, they can more accurately capture relationships in the development data. However, increased complexity can lead to overfitting, where the model captures noise rather than true population-level associations, resulting in poor performance on external data. Conversely, overly simple models may underfit, failing to capture important relationships. Regularization encompasses various techniques used during model development to limit model complexity and reduce overfitting.

### Bias

Bias in model development can arise from several sources, including nonrepresentative sample populations, inadequate sample sizes, or inappropriate statistical methods. Assessing the performance of a prediction model often focuses on overall metrics, such as a single c-statistic or calibration plot. However, assessing performance across key demographic groups, including sex, race, and ethnicity is essential to evaluate model fairness [8] and is recommended in recent reporting guidelines [9]. This approach helps prevent a model's use inadvertently perpetuating disparities in healthcare.

### Inadequate model validation

Validation is a critical step in the development of clinical prediction models. Internal validation assesses the model's performance on the development data. Simple assessment of the model on the development data (apparent performance) will likely give “optimistic” estimates of its discrimination and calibration. Methods such as cross-validation and bootstrapping can be used to adjust for this optimism, providing more realistic estimates of how the model would perform on unseen data. External validation involves testing the model on independent data, and is essential to confirm a model's robustness and applicability to various populations.

### Small sample sizes

Models developed on small datasets are prone to overfitting, where the model captures noise rather than the underlying clinical signal. This results in poor generalization to new data. Ensuring sufficient sample sizes during model development is crucial to produce a reliable and stable model. Recent publications and software can assist researchers in sample size calculations to determine the appropriate number of observations needed for model development [10] and validation [11]. Modelling practice has shown some improvements over time with sample sizes for the POSSUM score [12] (fewer than 1500) compared to American College of Surgeons (ACS) United States National Surgical Quality Improvement Program (NSQIP) [13] (1.4 million) as an example (see below).

Understanding performance metrics of perioperative risk models and their limitations is important for evaluating their effectiveness in clinical practice. In the following section, we explore several commonly used risk scores, evaluating their design, application, and performance.

## CURRENT PERIOPERATIVE SCORES

Common perioperative risk scores [1] are outlined below.

### American Society of Anesthesiologists score

The American Society of Anesthesiologists (ASA) score is an assessment tool that categorizes patients into six classes based on their preoperative physical health. The score was originally devised in 1941 as a way to communicate patient operative risk and to allow standardized surgical outcomes [14]. It has been revised on several occasions and in 1963 the five point scale most commonly known was first reported [15]. There is now an additional category for patients in whom death has been diagnosed through neurological criteria whose organs are being removed for donation purposes. The ASA score is simple to use and widely adopted due to its ease of application. However, its subjective nature can lead to significant inter-rater variability [16]. In a review of seven studies it had a median *c*-statistic of 0.77 (range 0.59–0.93) for perioperative mortality (with serious risk of bias) [1].

### Physiological and operative severity score for the enumeration of mortality and morbidity and Portsmouth variant

#### POSSUM

The POSSUM (Physiological and Operative Severity Score for the Enumeration of Mortality and Morbidity) score was originally developed by Copeland and colleagues in 1991 to assess quality of care and provide a scoring system for surgical audit [12]. It was developed on data from 1,372 patients who underwent elective or emergency general surgery from August 1988 to February 1989 in Liverpool, UK. POSSUM calculates a risk score based on 12 physiological and six operative variables. The original POSSUM model has been criticized for overestimating mortality rates in low-risk populations [17,18]. In a review of 13 studies, it had a median c-statistic of 0.82 (range 0.47–0.95) for perioperative mortality (with serious risk of bias) [1].

#### P-POSSUM (Portsmouth-POSSUM)

P-POSSUM is an adaptation of POSSUM, incorporating the same variables as POSSUM but modifying the weighting of these variables to improve calibration [19]. P-POSSUM consequently offers better calibration compared to the original POSSUM, including the low-risk patients in whom risk was overestimated by the original POSSUM score [18]. However, like POSSUM, it requires extensive data collection, which can limit its practicality in certain clinical settings. Discrimination appeared similar to POSSUM in a review of 18 studies (median c-statistic 0.81, range 0.56–0.94). Across 10 studies, P-POSSUM was better calibrated then POSSUM (calibration slope 1.03 vs. 0.86, respectively) [1].

#### The surgical outcome risk tool

The Surgical Outcome Risk Tool (SORT) was developed as a practical preoperative risk stratification tool to predict 30-day mortality following noncardiac surgery in adults [2]. SORT was derived from a large, prospective cohort study conducted by the National Confidential Enquiry into Patient Outcome and Death (NCEPOD), involving over 16,000 patients across multiple UK hospitals. The tool is based on a logistic regression model incorporating six preoperative variables. The SORT model demonstrated good predictive accuracy with a c-statistic of 0.91 in the validation cohort. In a comparison with POSSUM, P-POSSUM, the SORT score had the highest discriminative ability (*c*-statistic 0.922). However all models over-predicted risk, with SORT displaying calibration drift over time towards overprediction [20].

A 2020 update to the SORT tool introduced clinician-estimated risk into the model [21]. The updated SORT demonstrated that the predictive accuracy of the tool improved when clinician-estimated risk was combined with the original six variables [*c*-statistic 0.92, 95% confidence interval (CI) 0.90–0.94 vs. 0.90, 95% CI: 0.88–0.92].

#### National Emergency Laparotomy Audit score

The National Emergency Laparotomy Audit (NELA) was established in 2012 in the United Kingdom (UK) to improve perioperative care through providing high quality comparative data [22]. The NELA Calculator was developed for post hoc risk adjustment across cohorts from different hospitals but is increasingly used for patient-level mortality risk prediction in patients undergoing emergency laparotomy. It is recognized in international guidelines [23] and has been shown to have better discriminatory power for mortality after emergency laparotomy than the P-POSSUM score (0.818 vs. 0.769) [24] and the ACS NSQIP score (see below—0.83 vs. 0.80) [25].

#### American College of Surgeons United States National Surgical Quality Improvement Program

The NSQIP, developed in the 1990s, collects data on preoperative risk factors, perioperative care, and 30-day outcomes to benchmark surgical performance and reduce complications [26]. In 2013, the ACS developed the NSQIP Surgical Risk Calculator (SRC) using data from 1.4 million patients from 393 hospitals [13].

The ACS NSQIP risk calculator uses 21 preoperative predictors and goes beyond most other scores by providing risk estimates for multiple postoperative complications including venous thromboembolism, pneumonia, and renal failure. Across eight studies, it had a median c-statistic of 0.83 (range 0.62–0.97) for perioperative mortality (with serious risk of bias) [1].

## RECENT ADVANCES

### Understanding uncertainty

A recent model incorporated candidate variables from the NELA dataset, and estimates the distribution of predicted risks for a patient [27]. The RUNE (risk of death is uncertain in emergency laparotomy) model highlights the uncertainty over the risk of death due to the imprecision (i.e. random error) and the uncertainty introduced by prospective imputation of missing values in its mortality risk estimate, producing an individualized degree of uncertainty in predicted mortality risks.

### Incorporating ECG waveforms

A recent study developed and validated a deep-learning algorithm (PreOpNet) to predict 30-day postoperative mortality using 12-lead ECG waveform data from preoperative ECGs performed within 30 days of surgery [28^▪^]. The deep-learning algorithm predicted postoperative mortality in an external validation cohort with a c-statistic of 0.83, and MACE events with a *c*-statistic of 0.77. Although the PreOpNet study underwent external validation, it failed to assess the external validation performance of a comparator model, instead comparing it to the PreOpNet model performance in a held-out subsample of the development data, placing the comparator at a disadvantage.

### Large language models

Large language models (e.g. ChatGPT) are a type of machine learning model designed to understand, generate, and manipulate language. They are trained on large amounts of text data and use deep learning techniques often based on transformer architecture [29].

A recent paper evaluated the performance of a large language model in predicting perioperative risk and outcomes using electronic health records [30^▪^]. The model showed moderate to strong performance in predicting binary outcomes, including ICU admission and hospital mortality. However, performance was lower for duration-related outcomes such as ICU and hospital length of stay. This interesting study highlights the potential utility of these models in clinical workflows but underscores the need for further development.

## FUTURE DIRECTIONS

The future of perioperative risk prediction may lie in leveraging high-dimensional dynamic data and machine learning (ML) techniques. Although the boundary between ML and “traditional” statistical techniques is unclear [31], ML methods can often handle large amounts of data and complex interactions between variables, uncovering patterns that traditional statistical methods might miss. The integration of ML into perioperative medicine presents both significant opportunities and challenges. One concern is the propagation of biases through ML models, which can arise from biased training data or flawed algorithmic processes. These biases have the potential to exacerbate existing disparities in healthcare, particularly for marginalized populations, by influencing risk assessments and treatment decisions. Therefore, it is essential to ensure that ML systems are developed and implemented with rigorous oversight and transparency, and validated in important demographic subgroups [32].

### Data integration

Integrating diverse data sources, such as electronic health records (EHRs), imaging data, and genetic information, has the potential to enhance the predictive power of perioperative risk models. Machine learning algorithms can process and analyse these heterogeneous data types, identifying patterns and relationships that may not be apparent with traditional methods. For example, combining clinical data with genomic information could lead to more personalized risk predictions and targeted interventions, with our recent examples from oncology literature [33].

### Dynamic prediction

Dynamic prediction refers to a model's ability to continuously update its predictions in real-time as new data becomes available. Specifically, these predictions are not only made once at the start (preoperatively) but are continually adjusted using data from different stages of the surgical process. By integrating both preoperative and intraoperative time-series data, models can potentially provide more accurate predictions of complications as a patient's condition changes during the surgery. One recent study aimed to improve the prediction of postoperative complications using a dynamic deep learning model incorporating both preoperative and intraoperative data. A model processing physiological time series data outperformed those using summary preoperative and intra-operative data [34^▪^].

### Continuous learning

Prediction models have the potential to continuously learn and improve over time as new data becomes available. This continuous learning capability allows models to adapt to changing clinical practices and patient populations, maintaining their performance and relevance. It has been widely demonstrated that risk tools benefit from temporal updates and recalibration within target populations to produce meaningful absolute risks [20]. Implementing systems that allow for ongoing model updates and validation may ensure that perioperative risk models remain up to date, however would be practically challenging, for example through a real-time data Health Level 7 (HL7) feed from the hospital information system [35]. With some promising examples in radiology [36], these methods may extend to wider clinical data in the future.

### Optimal implementation

Successful implementation of complex models in healthcare requires a focus on qualitative factors and the principles of implementation science. This will improve integration into clinical workflows, addressing usability, clinician engagement, and real-world applicability. Recent work has explored this in the context of perioperative risk and recognized key themes [37], including the need for trust and interpretability in models, a user-friendly interface with visual representations, actionable insights on modifiable risks, and seamless integration into existing electronic health record systems for efficient use in practice

## CONCLUSION

Perioperative risk scores play an important role in surgical care but must be interpreted in the context of their limitations and often require ongoing refinements to maintain their relevance in evolving clinical environments. The integration of machine learning offers promising opportunities to improve predictive accuracy by incorporating multidimensional data. Future research should focus on developing interpretable, reliable, and generalizable models that integrate diverse data sources and continuously learn from new information [37].

## Acknowledgements

*None.*

### Financial support and sponsorship

*JB, OR, and PW were supported by the National Institute for Health Research (NIHR) Oxford Biomedical Research Centre (BRC).*

### Conflicts of interest

*None.*

## REFERENCES AND RECOMMENDED READING

Papers of particular interest, published within the annual period of review, have been highlighted as:

▪ of special interest
▪▪ of outstanding interest
